# Dietary Leucine and Total Polyphenol Intake and Their Associations with Sarcopenia Indicators Among Older People Participating in the Program for Complementary Food in Older People (PACAM) in the Metropolitan Region, Santiago de Chile

**DOI:** 10.3390/nu18142237

**Published:** 2026-07-09

**Authors:** Edson Bustos-Arriagada, Migdalia Caridad Arazo-Rusindo, Jorge Calderón-Herrera, Francisco Pérez-Bravo, Oscar Castillo-Valenzuela, Jorge Barros-Velázquez, María Salomé Mariotti-Celis

**Affiliations:** 1 Faculty of Medicine, Nutrition and Dietetics School, Universidad Finis Terrae, Pedro de Valdivia 1509, Providencia, Santiago 7501015, Chile; edsonbustos@uft.cl (E.B.-A.); jcalderonh@uft.edu (J.C.-H.); ocastillo@uft.cl (O.C.-V.); 2 Department of Analytical Chemistry, Nutrition and Food Science, Food Technology Division, School of Veterinary Sciences, University of Santiago de Compostela, Campus Lugo, 27002 Lugo, Spain; jorge.barros@usc.es; 3 Escuela de Nutrición y Dietética, Facultad de Salud, Universidad Santo Tomás, Avda. Ejército Libertador 146, Santiago 8370003, Chile; marazo@santotomas.cl; 4 Institute of Nutrition and Food Technology (INTA), University of Chile, Santiago 7830489, Chile; fperez@inta.uchile.cl; 5 Nutrigenomics Laboratory, Nutrition Department, Faculty of Medicine, University of Chile, Santiago 7830489, Chile

**Keywords:** sarcopenia, leucine, total polyphenols, older people, PACAM

## Abstract

Background and Objective: Sarcopenia is a prevalent condition among older people that significantly compromises functional capacity and quality of life. This cross-sectional study examined the associations between leucine and total polyphenol intake and indicators of sarcopenia in 181 older people from the Metropolitan Region of Chile enrolled in the PACAM program. Methods: Dietary intake was assessed using three non-consecutive 24-h dietary recalls. Leucine intake was estimated using Food Processor II software and compared with a 3 g/day anabolic threshold, while total daily polyphenol intake (mg/day) was calculated from Phenol-Explorer content data. Both dietary variables were examined in relation to sarcopenia status (presence/absence) as defined by the established European Working Group on Sarcopenia in Older People (EWGSOP2) diagnostic criteria. Statistical analyses included Mann–Whitney U tests for continuous variables, chi-square tests for categorical variables, and multivariable logistic regression models, with results expressed as odds ratios (ORs) and 95% confidence intervals (CIs); significance was set at *p* < 0.05. Results: The median intakes of leucine and total polyphenols were 2.2 g/day and 499.33 mg/day, respectively, with total polyphenols derived mainly from fruits and vegetables. No significant associations were observed between leucine or total polyphenol intake and sarcopenia status, either in absolute terms or when categorized by the anabolic thresholds (≥3 g/day and ≥1000 mg/day, respectively). In multivariable logistic regression models adjusted for age, BMI, total protein and total energy intake, neither leucine nor total polyphenol intake showed independent associations with sarcopenia. Conclusions: In this sample of older Chilean people, habitual intake of leucine and total polyphenols was not associated with sarcopenia status, suggesting that current intake levels may not be sufficient to produce measurable effects on muscle health under real-world conditions.

## 1. Introduction

Population aging is one of the major public health challenges of the 21st century, given its rapid progression and its clinical, functional, and socioeconomic implications [1]. In Chile, it is estimated that by 2050, nearly 30% of the population will be aged 60 years or older, underscoring the need to develop effective strategies to address chronic diseases and age-related conditions [2,3].

Among these conditions, sarcopenia stands out for its progressive loss of muscle mass, strength, and function [4,5]. Its etiology is multifactorial and involves chronic low-grade inflammation, mitochondrial dysfunction, hormonal changes, and, importantly, inadequate nutrition [6,7,8]. Insufficient intake of protein and essential amino acids, particularly leucine, as well as dietary antioxidants, has been associated with an increased risk of sarcopenia [9,10].

Leucine, one of the branched-chain amino acids (BCAAs), has demonstrated a direct anabolic effect on muscle protein synthesis by activating the mTORC1 signaling pathway [11,12]. Recent clinical studies have shown that leucine supplementation, alone or in combination with other nutrients, improves muscle strength, muscle mass, and physical performance in older people [11,13,14].

Polyphenols, bioactive compounds with antioxidant and anti-inflammatory properties, have also shown beneficial effects in preserving muscle mass and function by modulating systemic inflammation and oxidative stress, as well as signaling pathways associated with muscle anabolism, such as mTORC1 and AMPK [15,16,17]. Recent studies indicate that a daily intake of at least 1000 mg of polyphenols is associated with greater handgrip strength, improved physical performance, and a lower risk of sarcopenia in older people [17,18].

In addition, emerging evidence suggests a synergistic interaction between leucine and polyphenols. By attenuating oxidative stress and chronic inflammation, polyphenols may enhance the anabolic response induced by leucine, thereby supporting muscle protein synthesis [19,20]. This interaction could be particularly relevant in older people, in whom both nutrients may act through complementary mechanisms to help preserve muscle mass and function.

In Chile, the Program for Complementary Food in Older People (PACAM) provides fortified instant foods to older people at the country’s public health centers, including powdered soups formulated from cereal and legume ingredients and powdered dairy-based beverages, both enriched with vitamins and minerals. However, previous studies have reported low acceptability and limited effectiveness in relation to relevant nutritional parameters [21,22]. A recent study by the same research group also found no significant differences in sarcopenia indicators between older people with adequate and inadequate leucine intake who regularly consumed PACAM foods [23]. These findings suggest that the current portfolio of fortified products may be insufficient to improve muscle mass, strength, and functional outcomes in this vulnerable population.

According to the most recent national estimate of total polyphenol intake in Chile, based on the 2010–2012 National Food Consumption Survey (ENCA), the average daily intake for the adult population, including older people, is 979.5 ± 695.5 mg/day [24]. However, these data are derived from dietary patterns that may not reflect current consumption habits. In comparison, international studies focused specifically on older people have reported lower mean intakes, ranging from 332.7 mg/day [25] to 414.2 mg/day [26]. Updated, population-specific data are needed to accurately assess polyphenol intake among older people in Chile, especially given the potential synergistic effects of polyphenol intake and leucine supplementation on muscle health and sarcopenia.

This study aims to provide local evidence on the relationships among dietary leucine intake, polyphenol intake, and sarcopenia-related parameters in Chilean older people receiving PACAM, and to conduct qualitative and quantitative analyses of polyphenol intake in this population.

## 2. Materials and Methods

### 2.1. Study Design & Population

This observational cross-sectional study was conducted within the framework of the FONDEF Project (ID17AM0018). The sampling frame comprised 209 government-funded food distribution centers participating in the Program for Complementary Food in Older People (PACAM) in Chile’s Metropolitan Region. Older people were recruited from these centers using non-probabilistic convenience sampling. Participants were eligible for inclusion if they were aged ≥ 60 years, enrolled in the PACAM program, and able to provide informed consent. Participants were excluded if they had incomplete dietary recall data or missing key variables required for the analysis. A total of 181 older people met the eligibility criteria and were included in the final analytical sample, which was used to examine the association between dietary leucine and polyphenol intake and sarcopenia status [23].

### 2.2. Anthropometric Characterization and Sarcopenia Assessment

Body weight was measured to the nearest 0.1 kg using a calibrated digital scale (SECA 803, Hamburg, Germany, precision 0.1 kg), with participants wearing light clothing and no shoes. Height was measured to the nearest 0.1 cm using a portable stadiometer (SECA 213, Hamburg, Germany, precision 0.1 cm). Body mass index (BMI) was calculated as weight (kg) divided by height squared (m^2^), and nutritional status was classified according to the criteria established by the Chilean Ministry of Health for older people.

Sarcopenia status was defined according to the revised EWGSOP2 criteria (2019) [4]. Muscle strength was assessed using handgrip strength (HGS), measured with a hand dynamometer (Lafayette Hydraulic Hand Dynamometers model J00105, Lafayette Instrument Company, Lafayette, IN, USA) on the dominant hand, and the highest value was recorded. Low muscle strength was defined using established cut-off points (<27 kg for men and <15 kg for women). Muscle mass was estimated using the appendicular skeletal muscle mass index (SMI), calculated based on validated anthropometric predictive equations. Low muscle mass was defined using sex-specific cutoffs (<7.45 kg/m^2^ for men and <5.88 kg/m^2^ for women). Physical performance measures (e.g., gait speed or the Timed Up and Go test) were not included because of the field-based nature of data collection in community settings. Sarcopenia was defined as the presence of both low muscle strength and low muscle mass. In the present study, sarcopenia was treated as a binary variable (presence/absence).

### 2.3. Dietary Assessment

Food consumption was estimated using three non-consecutive 24-h dietary recalls (24-hDRs), including two weekdays and one weekend day, to account for intra-week variability and improve the representativeness of habitual intake. All recalls were administered by trained interviewers using standardized neutral probing questions based on the Multiple Pass Method (MPM) [27].

To enhance the accuracy of portion-size estimation, interviews were supported by the validated graphic portion booklet developed by the Universidad de Chile and MINSAL [28]. Interviewers underwent standardized training conducted by certified nutritionists, who simultaneously supervised fieldwork to ensure consistency and quality control. The homemade measurements reported in the dietary recalls were converted into grams (g) and milliliters (mL) using standardized conversion references [29].

As part of the dietary assessment, consumption of foods provided by the PACAM was also documented within the 24-h dietary recalls, allowing its contribution to total dietary intake, including leucine and total polyphenol intake, to be incorporated under habitual consumption conditions.

### 2.4. Estimated Daily Intake of Leucine, Protein, Energy and Polyphenols

The daily dietary intake of leucine, protein, energy and polyphenols was estimated from the leucine, protein, energy and total polyphenol contents of each food item, along with individual food consumption data derived from 24-h dietary recall questionnaires administered to study participants.

Leucine, total protein, and total energy intake were estimated using the Food Processor II version 7.9 nutritional analysis software (ESHA Research, Salem, OR, USA) and subsequently expressed as total daily intake (g/day). The estimated intake was then compared with the recommended per-meal dose (RPMD) of 3 g/day [30]. The cut-off point for leucine (≥3 g/day) was based on evidence from intervention studies suggesting that approximately 3 g of leucine per meal may be required to stimulate muscle protein synthesis; however, this value was used here as an exploratory threshold for daily intake.

Polyphenol intake was estimated using the following equation:

$$Estimated\;daily\;polyphenol\;intake=\sum_{i=1}^{n}C_{i}Cr_{i}$$

where *C_i_* represents the mean content of total polyphenols in food *i* (mg of total polyphenols g^−1^ of food), *Cr_i_* corresponds to the daily consumption of food *i* (g day^−1^) as determined by the 24-h dietary recall, and *n* is the number of foods included in the calculation. The values for total polyphenol content (*C_i_*) were obtained from the Phenol-Explorer database, which compiles standardized data on polyphenol content in foods derived from published analytical studies [31]. The cut-off point for total polyphenol intake (≥1000 mg/day) was based on values reported in observational studies as indicative of relatively high intake, and was used for categorization rather than as a recommended intake level.

### 2.5. Statistical Analysis

Descriptive statistics were calculated for all variables. Continuous variables were assessed for normality and are presented as medians and interquartile ranges (IQRs), as most variables did not follow a normal distribution. Differences between participants with and without sarcopenia, and between estimated daily leucine and total polyphenol intake, were evaluated using the Mann–Whitney U test for continuous variables and the chi-square test for categorical variables.

Multivariable logistic regression analyses were conducted to evaluate the independent associations between dietary intake and sarcopenia status. Sarcopenia was coded as 0 (sarcopenic) and 1 (non-sarcopenic). Age, BMI, total energy intake, and total protein intake were modeled as continuous variables. Separate models were fitted for leucine intake (≥3 g/day) and total polyphenol intake (≥1000 mg/day) to avoid potential collinearity between dietary variables. All models were adjusted for age, BMI, total energy intake, and total protein intake to account for overall dietary exposure. Odds ratios (ORs) and 95% confidence intervals (CIs) were calculated. Model fit was evaluated using the Hosmer–Lemeshow goodness-of-fit test, and explained variance was estimated using the Nagelkerke R^2^ statistic.

A two-tailed *p*-value < 0.05 was considered statistically significant. All analyses were performed using IBM SPSS Statistics version 27.0.1.0, IBM Corp., Armonk, NY, USA.

### 2.6. Ethical Considerations

The study was conducted in accordance with the principles of the Declaration of Helsinki. The study protocol was reviewed and approved by the Ethics Committee of the Institute of Food Nutrition and Technology (INTA) of the University of Chile (Project identification code 20/2017). All participants received detailed information about the study objectives and procedures and provided written informed consent prior to participation.

## 3. Results

### 3.1. Participant Characteristics

The sample comprised 181 older people enrolled in the PACAM program, 80% of whom were women and 20% were men. A total of 45.9% were older than 75 years. Regarding nutritional status, 13.3% were underweight, 44.7% had a normal weight, 28.7% were overweight, and 13.3% were obese, with no significant differences by sex.

### 3.2. Estimated Leucine, Total Protein, Total Energy, and Total Polyphenol Intake

The majority of participants (80.7%) did not meet the RPMD of leucine, with no significant sex differences. The median daily intake of total protein and total energy were 46.53 g/day and 1162.43 kcal/day, respectively.

The median daily intake of total polyphenols (TPC) was 499.33 mg/day, below the 1000 mg/day threshold previously associated with positive effects on muscle anabolism. Overall, 64.1% of the sample reported consuming PACAM-provided foods, with women representing the majority of consumers (Table 1).

**Figure 1:**
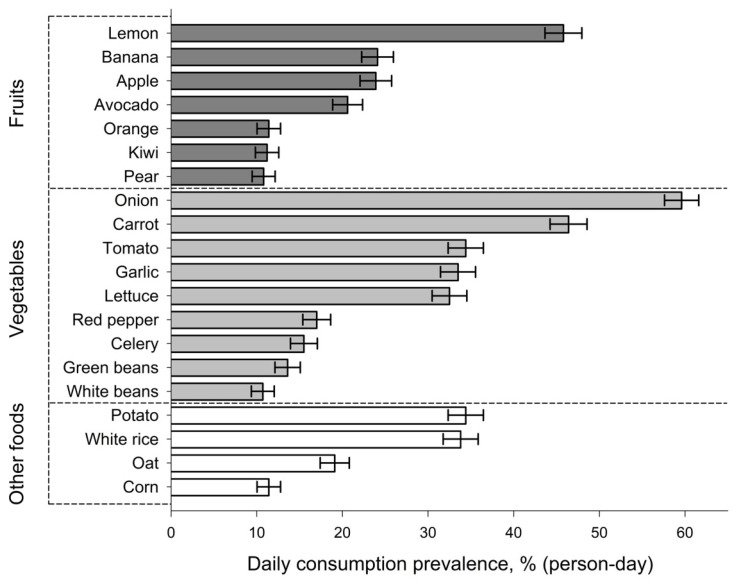
Top 20 polyphenol-containing foods by daily consumption prevalence (person-days) based on three 24-h dietary recalls (24-hDR) in 181 older people.

**Figure 2:**
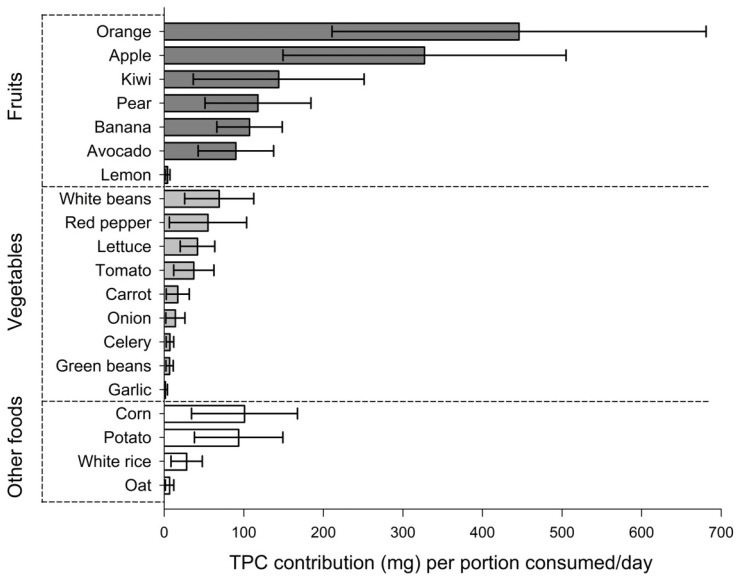
Average of TPC contribution (mg) per portion consumed/day of the 20 most frequently reported foods in 181 older people (n = 181).

The 20 most frequently consumed foods were identified from three 24-h dietary recalls (Figure 1). Among vegetables, onion, carrot, potato, and tomato were most commonly reported, while lemon, banana, apple, and avocado predominated among fruits. Polyphenol contribution per daily serving was calculated based on these foods (Figure 2). Fruits were the primary polyphenol contributors, with orange, apple, kiwi, pear, banana, and avocado representing the highest per-serving values. Among the vegetables, potato, red bell pepper, lettuce, and tomato had the highest polyphenol content per serving.

When analyzing the effects of sociodemographic variables, leucine intake, sarcopenia, nutritional status, and PACAM consumption on total polyphenol intake (Supplementary Material, Table S1), a homogeneous distribution was observed, with no significant differences by sex, age, nutritional status, presence of sarcopenia, or leucine RPMD compliance.

### 3.3. Sarcopenia Prevalence and Comparisons Between Groups

The overall prevalence of sarcopenia was 17.1%, with a significantly higher proportion in women than in men (*p* = 0.026). Subsequently, the effects of age, total daily polyphenol intake, total daily leucine intake, nutritional status, and PACAM consumption were evaluated in older people with sarcopenia (n = 31) and without sarcopenia (n = 150). Medians and interquartile ranges are presented, along with *p*-values to assess statistical significance (Table 1).

Participants with sarcopenia were significantly older than those without (*p* = 0.016), and this difference remained significant among those older than 75 years (*p* = 0.031), suggesting a higher frequency of sarcopenia at more advanced ages. BMI differed significantly between groups (*p* < 0.001), with underweight being more frequent among participants with sarcopenia (*p* = 0.046), while no differences were observed in the overweight or obesity categories. PACAM consumption did not differ significantly between groups.

No significant differences were found in leucine intake, either in absolute terms or by RPMD compliance. The same pattern was seen in both total protein and total energy intake. Likewise, there were no significant differences in daily total polyphenol intake between participants with and without sarcopenia (*p* = 0.241). When stratified by the ≥1000 mg/day threshold, no significant differences were observed between groups, either among those consuming < 1000 mg/day (*p* = 0.225) or among those consuming ≥1000 mg/day (*p* = 0.096). Overall, these findings indicate that total polyphenol intake was not significantly associated with sarcopenia in this sample.

### 3.4. Multivariable Logistic Regression Analyses

To evaluate the independent associations between dietary intake and sarcopenia status, multivariable logistic regression analyses were performed (Table 2). All models were adjusted for age, BMI, total energy intake, and total protein intake. Age was inversely associated with non-sarcopenic status (OR = 0.91; 95% CI: 0.83–0.98; *p* = 0.020). Conversely, BMI was positively associated with non-sarcopenic status (OR = 1.31; 95% CI: 1.14–1.50; *p* < 0.001). Total energy intake (OR = 0.999; 95% CI: 0.997–1.001; *p* = 0.400) and total protein intake (OR = 1.02; 95% CI: 0.97–1.07; *p* = 0.503) were not significantly associated with sarcopenia status.

Model fit was adequate in both models (Hosmer–Lemeshow test *p* > 0.05), and explained variance was approximately Nagelkerke R^2^ = 0.256.

When leucine intake (≥3 g/day) was included in the model, no statistically significant association was observed (OR = 0.63; 95% CI: 0.23–1.80; *p* = 0.381). Similarly, total polyphenol intake (≥1000 mg/day) was not independently associated with sarcopenia status (OR = 2.70; 95% CI: 0.20–42.93; *p* = 0.433). Model fit was adequate across all models (Hosmer–Lemeshow test *p*-values > 0.05), with an explained variance of approximately 0.256 (Nagelkerke R^2^).

## 4. Discussion

### 4.1. Participant Characteristics and Sarcopenia Prevalence

The proportion of participants with sarcopenia in this study is consistent with prevalence estimates previously reported in community-dwelling older people [5,32]. Sarcopenia was assessed according to the EWGSOP2 criteria using measures of muscle strength and estimated muscle mass; however, physical performance was not evaluated because of the field-based nature of data collection. Future studies incorporating screening tools and functional performance assessments may provide a more comprehensive characterization of sarcopenia and help further clarify its relationship with dietary intake.

### 4.2. Dietary Intake Patterns and Food Sources of Polyphenols

In the context of accelerated population aging in Chile and persistent inequalities in functional health [1,2,3], identifying dietary factors associated with healthy aging remains a public health priority. While PACAM represents an important nutritional support strategy for older people, the present findings suggest opportunities to further strengthen dietary quality by promoting foods naturally rich in bioactive compounds and high-quality protein.

The analysis of dietary sources provides additional context, as fruits and vegetables were identified as the main contributors to total polyphenol intake. This finding is consistent with international data reported in Phenol-Explorer and related databases [33]. Moreover, evidence from dietary pattern research indicates that diets rich in fruits and vegetables are associated with better physical performance and a lower risk of sarcopenia [34,35,36,37]. These associations likely reflect the combined effects of multiple nutrients and bioactive compounds acting within the overall dietary matrix, highlighting the importance of considering dietary patterns rather than isolated nutrients when evaluating nutritional determinants of muscle health.

### 4.3. Estimated Leucine, Total Protein, Energy, and Total Polyphenol Intakes

The majority of participants did not meet the proposed reference intake for leucine, and the median daily intake of total polyphenols was below the threshold previously associated with positive effects on muscle anabolism. Although these findings suggest a relatively low exposure to both compounds in this population, the corresponding cut-off values should be interpreted with caution, as they are not based on established dietary recommendations. In particular, the leucine threshold derives primarily from per-meal intervention studies rather than habitual daily intake.

Despite these limitations, characterizing habitual dietary intake provides valuable translational information. Quantifying leucine and polyphenol intake helps establish baseline exposure levels that may inform the design of nutritional interventions and functional foods for older people. In addition, the relatively low median intakes of protein and energy observed in the study population provide an important context for interpreting muscle health outcomes, as overall dietary adequacy may influence the effects of specific nutrients and bioactive compounds.

For leucine, formulation considerations are equally important. Although central to anabolic signaling, higher concentrations may impart bitterness, potentially limiting palatability and adherence [38]. Baseline intake data, therefore, contribute to rational product formulation by balancing physiological efficacy with sensory feasibility.

In the case of polyphenols, dose considerations are particularly relevant, as biological responses are highly context-dependent and strongly influenced by bioavailability, metabolic transformation, and redox status [39]. While moderate dietary exposures have been associated with beneficial effects, excessive concentrations may induce pro-oxidant activity under specific physiological conditions [40,41]. Defining population-level intake ranges therefore provides an essential reference for identifying physiologically appropriate fortification levels and for ensuring that enrichment strategies remain within biologically meaningful and safe limits.

### 4.4. Sarcopenia Status and Dietary Intake Comparisons

Daily leucine intake did not differ between participants with and without sarcopenia. Leucine intake was analyzed as an independent dietary variable, given its recognized role in regulating muscle protein synthesis and its anabolic benefits in controlled intervention studies [13,14]. However, observational assessments of total intake may not capture critical determinants such as protein quality, meal distribution, or metabolic responsiveness in older people. Likewise, neither total protein intake nor total energy intake differed by sarcopenia status, suggesting that overall dietary quantity alone may not adequately explain variations in muscle health in this population.

Similarly, no significant associations were observed between total polyphenol intake and sarcopenia status. Experimental evidence supports the role of polyphenols in modulating oxidative stress and inflammatory pathways relevant to muscle preservation [17,18,19,42,43]. However, most of the beneficial effects reported in experimental and supplementation studies are derived from controlled conditions with defined doses, which may not be directly comparable to habitual dietary intake. These findings highlight the need to differentiate between supplementation and dietary exposures in future research.

Taken together, these results suggest that under habitual dietary conditions, overall dietary quality may exert a greater influence on muscle health than isolated nutrient exposure. Proposed synergistic effects between leucine and polyphenols on anabolic signaling and oxidative balance [7,8,44,45] may depend on dose, bioavailability, and long-term dietary patterns rather than short-term intake estimates. In this sense, these interpretations should be considered in light of the cross-sectional design, which precludes causal inference. The relationship between habitual diet and muscle health likely reflects cumulative exposures and complex metabolic interactions over time, highlighting the need for longitudinal and interventional studies in older Chilean populations.

### 4.5. Associations Between Dietary Intake and Sarcopenia Status

Multivariable logistic regression models were used to assess the independent associations between dietary variables and participant characteristics and sarcopenia status. Neither leucine intake (≥3 g/day) nor total polyphenol intake (≥1000 mg/day) was significantly associated with sarcopenia after adjustment for age, BMI, total protein intake, and total energy intake. Similarly, total protein and total energy intakes were not independently associated with sarcopenia, suggesting that neither specific nutrient exposure nor overall dietary intake explained the observed differences in sarcopenia status within this population.

Although the prevalence of sarcopenia resulted in unequal group sizes, a common feature of observational studies, the regression analyses yielded consistent estimates across model specifications. While some confidence intervals were relatively wide, particularly for polyphenol intake, this likely reflects the limited number of participants with higher intake levels rather than a consistent pattern of association. Model fit was adequate across all specifications (Hosmer–Lemeshow *p* > 0.05; R^2^ = 0.256), supporting the robustness of the analytical approach despite the absence of significant dietary associations. These findings suggest that factors beyond the evaluated dietary exposures may exert a greater influence on sarcopenia status in this population.

Accordingly, age and BMI emerged as the only variables independently associated with sarcopenia status in the adjusted models. However, the observed association with BMI should not be interpreted as a direct protective effect, as BMI does not distinguish between fat and muscle mass and may reflect broader aspects of body composition rather than a causal mechanism. This distinction is particularly relevant in older people, among whom preserved body mass may coexist with reduced muscle quality and function. These findings reinforce the multifactorial nature of sarcopenia and suggest that the relationship between habitual diet and muscle health may not be adequately captured by isolated dietary exposures or short-term intake estimates.

### 4.6. Strengths and Limitations

This study provides insight into habitual dietary leucine and total polyphenol intake and their relationship with sarcopenia in community-dwelling older people participating in the PACAM program. The dietary assessment strategy, based on three non-consecutive 24-h dietary recalls administered through the Multiple Pass Method, together with the inclusion of PACAM foods, represents a notable strength of the study. However, several considerations should be taken into account when interpreting these findings. Although using three non-consecutive 24-h dietary recalls strengthens estimates of habitual dietary intake, this approach may not fully capture long-term dietary patterns, particularly for total polyphenol intake, which exhibits substantial day-to-day variability. Although total protein and energy intake were included in the regression models, other factors such as physical activity, comorbidities, inflammatory status, and vitamin D levels were not available and may contribute to residual confounding. Finally, the cross-sectional design enables the evaluation of associations under real-world conditions but does not allow for causal inference. Although experimental studies suggest potential complementary effects of leucine and polyphenols on skeletal muscle metabolism, their interaction was beyond the scope of the present study and warrants further investigation in longitudinal and intervention-based research.

## 5. Conclusions

The present study shows that sarcopenia is a relevant condition among older Chilean people enrolled in PACAM, with a higher prevalence among women and individuals of more advanced age. No significant associations were observed between sarcopenia and habitual dietary intake of leucine or total polyphenols. Similarly, total protein and energy intakes were not independently associated with sarcopenia status in the adjusted models. However, the overall intake levels of both nutrients observed were lower than those commonly reported in intervention studies demonstrating anabolic or antioxidant effects.

In this population of community-dwelling older people, leucine and total polyphenol intake were not independently associated with sarcopenia status. These findings suggest that, under habitual dietary conditions, the contribution of individual dietary components to sarcopenia may be difficult to detect. Rather than isolated nutrient intake, broader aspects of dietary quality, overall dietary patterns, and lifestyle factors may play a greater role in preserving muscle mass and function during aging. Further studies using longitudinal and intervention-based designs are needed to clarify these relationships.

Despite the absence of significant associations, the present findings contribute to the characterization of dietary intake patterns among older Chilean people participating in PACAM. The relatively low intake of leucine and total polyphenols observed in this population provides a relevant baseline for future research and nutritional strategies aimed at supporting healthy aging.

## Figures and Tables

**Table 1:** Sarcopenia according to sociodemographic variables, polyphenol intake, leucine intake, protein intake, energy intake, nutritional status and PACAM consumption (n = 181).

Evaluated Parameters	Sarcopenia		*p*-Value ^c^
	Yes (n = 31)	No (n = 150)	
	Median (25th, 75th)	Median (25th, 75th)	
**Age (years)**	78.0 (73.0, 82.5)	75.0 (72.0, 79.0)	0.016
60–75	72.0 (72.0, 74.0)	72.0 (71.0, 74.0)	0.913
>75	82.0 (78.8, 86.3)	80.0 (77.0, 82.0)	0.031
**TPC daily intake (mg)**	464.99 (265.99, 785.69)	538.29 (298.77, 805.82)	0.241
<1000 ^a^	364.79 (257.87, 573.87)	475.56 (265.49, 664.22)	0.225
≥1000 ^a^	1523.36 (1152.22, 2088.14)	1211.23 (1136.84, 1342.22)	0.096
**Leucine daily intake (g)**	2.42 (1.31, 3.11)	2.14 (1.66, 2.80)	0.803
<RPMD ^b^	1.78 (1.28, 2.52)	1.91 (1.59, 2.37)	0.488
≥RPMD ^b^	3.61 (3.22, 4.08)	3.58 (3.27, 4.24)	0.668
**Total protein daily intake (g)**	46.72 (38.27, 51.75)	45.92 (36.53, 58.95)	0.636
**Total energy daily intake (kcal)**	1133.27 (992.61, 1285.51)	1164.68 (929.08, 1409.49)	0.741
**BMI (kg/m^2^)**	25.35 (21.54, 26.83)	27.72 (25.29, 30.42)	<0.001
Underweight	20.14 (18.24, 21.54)	21.72 (20.40, 22.75)	0.046
Normal weight	26.56 (24.74, 26.84)	25.67 (24.72, 26.88)	0.461
Overweight	28.98 (28.07, 29.08)	29.38 (28.74, 30.51)	0.193
Obesity	-	35.04 (33.23, 36.77)	-
**PACAM food consumption**	**% (n)**	**% (n)**	***p*** **-value ^d^**
Consumes Does not consume	19.0 (22) 13.8 (9)	81.0 (94) 86.2 (56)	0.297

TPC: total polyphenol content; ^a^ daily dose observed to promote muscle anabolism; ^b^ RPMD: recommended per-meal dose; BMI: body mass index; PACAM: Program for Complementary Food in Older People; ^c^ *p*-value for U Mann–Whitney between age, leucine, TPC protein and energy daily intake groups; ^c^ *p*-value for Kruskal–Wallis for BMI; ^d^ *p*-value for Chi-square between PACAM food consumption groups.

**Table 2:** Multivariable logistic regression models assessing the association between dietary intake and sarcopenia status in older people (n = 181).

Variable	OR	95% CI	*p*-Value
Age (years)	0.91	0.83–0.98	0.020
BMI (kg/m^2^)	1.31	1.14–1.50	<0.001
Total energy intake (kcal/day)	0.999	0.997–1.001	0.400
Total protein intake (g/day)	1.02	0.97–1.07	0.503
Leucine intake (≥3 g/day) ^1^	0.63	0.23–1.80	0.381
Polyphenol intake (≥1000 mg/day) ^2^	2.70	0.20–42.93	0.433

OR: odds ratio; CI: confidence interval; BMI: body mass index. Sarcopenia status was coded as 0 = sarcopenic and 1 = non-sarcopenic. Age and BMI were modeled as continuous variables. All models were adjusted for age, BMI, total energy intake, and total protein intake. ^1^ Estimated from a model including age, BMI, total energy intake, total protein intake, and leucine intake. ^2^ Estimated from a model including age, BMI, total energy intake, total protein intake, and polyphenol intake.

## Data Availability

All data analyzed in this study are available upon request to the corresponding author.

## Supplementary Materials

The following supporting information can be downloaded at: https://www.mdpi.com/article/10.3390/nu18142237/s1, Table S1: Polyphenol consumption according to sociodemographic variables, leucine intake, sarcopenia, nutritional status and PACAM consumption (n = 181).
