# Supplementary material for: Dietary Leucine and Total Polyphenol Intake and Their Associations with Sarcopenia Indicators Among Older People Participating in the Program for Complementary Food in Older People (PACAM) in the Metropolitan Region, Santiago de Chile
Source: Nutrients. 2026 Jul 9;18(14):2237. doi: 10.3390/nu18142237 (PMC13414719; doi:10.3390/nu18142237)
Supplement: Supplementary file 1 [file nutrients-18-02237-s001.zip › nutrients-4286783-supplementary.pdf]

Supplementary Material Table S1. Polyphenol consumption according to sociodemographic variables, leucine intake, sarcopenia, nutritional status and PACAM consumption (n=181)

**Groups**		**TPC daily intake (mg/día)**	
		Median (25th, 75th)	*p*-value
Gender	Male	394.15 (241.03, 834.79)	0.660
	Female	509.86 (273.53, 783.81)	
Age	60-75	493.99 (267.36, 798.11)	0.986
	> 75	509.86 (269.31, 778.20)	
Nutritional status	Underweight	511.94 (267.98, 860.90)	0.313
	Normal weight	453.32 (251.01, 761.53)	
	Overweight	545.15 (359.90, 797.76)	
	Obesity	541.85 (281.44, 741.16)	
Sarcopenia	Yes	464.99 (265.99, 785.69)	0.241
	No	538.29 (298.77, 805.82)	
PACAM foods	Consumes	475.56 (265.99, 801.53)	0.756
	Does not consume	511.94 (278.44, 745.48)	
Leucine daily intake (g)	< RDD [a]	483.29 (263.42, 785.43)	0.251
	≥ RDD [a]	526.50 (372.57, 797.57)	

TPC: total polyphenol content; PACAM: Program for Complementary Food in Older People; [a] RDD: recommended daily dose; *p*-value for U Mann–Whitney between age, gender, sarcopenia, PACAM foods, and leucine total daily groups; *p*-value for Kruskal-Wallis for nutritional status group.
